# Endoplasmic reticulum stress related IncRNA signature predicts the prognosis and immune response evaluation of uterine corpus endometrial carcinoma

**DOI:** 10.3389/fonc.2022.1064223

**Published:** 2023-01-04

**Authors:** Jun Chen, Licong Shen, Yongwen Yang

**Affiliations:** ^1^ Department of Infectious Diseases, Xiangya Hospital, Central South University, Changsha, China; ^2^ National Clinical Research Center for Geriatric Disorders, Xiangya Hospital, Central South University, Changsha, China; ^3^ Department of Gynecology, Xiangya Hospital, Central South University, Changsha, China; ^4^ Department of Clinical Laboratory, Xiangya Hospital, Central South University, Changsha, China

**Keywords:** ER stress, lncRNAs, uterine corpus endometrial carcinoma, immune infiltration, drug therapy

## Abstract

**Background:**

Endoplasmic reticulum (ER) stress is closely related to the occurrence, development and treatment of tumors. Recent studies suggest ER stress as a therapeutic strategy of choice for cancer. However, ER stress-related long non-coding RNA (lncRNA) predictive value in endometrial carcinoma (UCEC) remains to be further evaluated. The purpose of this study was to establish relies on the signature of ER stress-related lncRNA forecast to predict the prognosis of patients with UCEC.

**Methods:**

We downloaded the RNA expression profile dataset and matched clinical data from the Cancer Genome Atlas (TCGA) database, and applied univariate and multivariate Cox regression analysis to build predictive signature. Kaplan-meier method was used to evaluate overall survival (OS) and disease-free survival (DFS). Gene set enrichment analysis (GSEA) was used to study the functional characteristics. Single sample Gene set enrichment analysis (ssGSEA) was used to analyze the relationship between immune status and predicted signature. Correlations between the potential usefulness of treatment for UCEC patients and predictive signature were also analyzed.

**Results:**

We established a signature composed of eight ER stress-related lncRNAs (MIR34AHG, AC073842.2, PINK1AS, AC024909.2, MIR31HG, AC007422.2, AC061992.1, AC003102.1). The signature of ER stress-related lncRNA provided better diagnostic value compared with age and tumor grade, and the area under the receiver operating curve was 0.788. The overall and disease-free survival probability of patients in the high-risk group is lower than that in the low-risk group. GSEA indicated that the pathways were mainly enriched for cancer, immunity and reproduction related pathways. ss-GSEA shows that prediction signature and activation of dendritic cells, immature dendritic cells, T helper cells and immune status of the Treg are significantly related. High-risk groups may against PD - 1/L1 immunotherapy and JNK inhibitors VIII, Z.LLNle.CHO, DMOG and JNK. 9 l more sensitive.

**Conclusion:**

The ER stress signature can independently predict the prognosis of UCEC patients, and provide guidance for conventional chemotherapy and immunotherapy of UCEC patients.

## Background

Uterine corpus endometrial carcinoma (UCEC) is considered to be the second cancer and the main cause of death in female cancer patients due to its high recurrence rate (1). It is a kind of endometrial epithelial malignant tumor with high mortality and a serious threat to women’s health. The incidence of non-estrogen-dependent tumors is low, but the degree of malignancy is high and the prognosis is poor (2). Data from the International Federation of Obstetricians and Gynaecologists show a significant decline in survival in patients with advanced UCEC and metastatic endometrial cancer (3). The treatment of UCEC has progressed with the advent of immune checkpoint inhibitors. For UCEC, the use of dostarlimab (anti-programmed cell death protein 1 antibody) alone or a tyrosine kinase inhibitor combined with pembrolizumab for advanced, metastatic, or recurrent endometrial cancer holds promise (3). However, neoadjuvant therapy for UCEC remains complex and controversial (4). Therefore, searching for predictive markers of UCEC is of great significance to reveal the recurrence of UCEC and explore new precision therapeutic targets.

Endoplasmic reticulum (ER) stress in promoting tumor growth, tumor immune microenvironment and chemotherapy drug resistance plays an important role (5). The disorderly and rapid growth of cancer cells and the hypoxic and malnourished tumor environment lead to ER stress. ER stress induces UPR so that cells can survive under adverse microenvironmental conditions, thus promoting tumor progression (6,7). On the other hand, cancer cells may disrupt the endoplasmic reticulum balance of immune cells, thereby resisting the beneficial antitumor effects of immune cells. A variety of factors in the tumor microenvironment can induce endoplasmic reticulum stress in Treg, which is beneficial to the survival of tumor cells (6). Ablation of ER stress kinase PERK induces ptosis and type I interferon promotes antitumor T-cell responses (8). Sec62 protein secreted proteins in eukaryotic cells and membrane after binding protein translation of transhipment, through direct interaction with Sec61 channel regulating intracellular calcium homeostasis, and in the recovery process of compensation to make a decisive contribution to the cells of the ER stress (9). IRE1α is one of three ER transmembrane sensors for the unfolded protein response (UPR) activated in response to ER stress. IRE1α overexpression in malignant cells limits tumor progression by inducing anticancer immune responses (7). Therefore, ER stress-related genes may become potential targets for tumor immunotherapy.

Long non-coding RNAs (lncRNAs) are an influential new class of non-coding RNAs. Cancer cells can regulate UPR influence on lncRNA of endoplasmic reticulum stress levels to ensure their survival under adverse conditions (10). Nine ERS-related lncRNA signature components have excellent predictive performance in predicting the prognosis of breast cancer, and are significantly correlated with clinicopathological features (11). Amino acid restriction induces a long non-coding RNA UBA6-AS1 to regulate GCN2-mediated integrated stress response in breast cancer (12). The long non-coding RNA RP5-821D11.7 promotes the proliferation, migration and epithelial-mesenchymal transition of glioma and glioma stem cell-like cells (13). At present, there are few studies on ER stress-related lncRNAs, and studies on ER stress-related lncRNAs in UCEC have not been retrieved. In this study, we established a predictive signal based on ER stress-related lncRNAs; identified its value for prognosis, diagnosis, tumor immune infiltration, and chemotherapy response in UCEC patients; and performed internal validation. Then, we explored the underlying mechanism by gene enrichment analysis (GSEA).

## Materials and methods

### Patients and datasets

We from TCGA website (https://portal.gdc.cancer.gov/) to download the cancer genome atlas endometrial cancer (TCGA - UCEC) data set of STAR - RNA - seq data count as well as matching the clinical and prognostic data repository); the data of 427 patients with lncRNA expression values and survival time were obtained. We collected disease-free survival (DFS) data from the cBioPortal database (https://www.cbioportal.org/) for 529 UCEC patients. A total of 8763 ER stress-related genes were downloaded from Gene Cards (https://www.genecards.org/).

### Functional enrichment analysis of differentially expressed ER stress-related genes

We applied the false discovery rate (FDR)< 0.01 and | change log 2 times (FC) > 3 | as filter conditions for ER stress-related to differentially expressed genes (DEGs). We used Kyoto Encyclopedia of Genes and Genomes (KEGG) and Gene Ontology (GO) analyses in the “ggplot2” and “Heatmap” packages.

### Establishment of the ER stress-related lncRNA predictive signature

We performed “limma” package to quantitative correlation between lncRNA and ER stress-related genes. Using the correlation coefficient | | R2 > 0.2 and p< 0.05 as filter criteria, received 1599 ER stress related lncRNA with expression values. We first to get related to the prognosis of patients with UCEC ER stress-related lncRNA used univariate Cox regression analysis, then we multivariate Cox regression analysis to obtain the ER stress related lncRNA, lncRNA prediction features related to establish ER stress. Each UCEC patients risk score computation formula is as follows:


$$\text{Risk}\;\text{score}=\sum_{i+1}^{n}(Coef_{i}\times x_{i})$$

Coef indicates the coefficient value, and x indicates the expression level of selected ER stress-related lncRNAs.

### Establishment of nomogram

We combined the risk score with the clinicopathological variables of age and grade to build a nomogram that can predict the 1 -, 3 -, and 5-year survival probability of UCEC patients. We had to verify the calibration curve of the correlation between predicted survival rate and the actual survival.

### Functional enrichment analysis of the ER stress-related lncRNA predictive signature

UCEC patients were divided into low risk group and high risk group according to the median risk score. GSEA was applied to obtain the major enriched pathway genes. GSEA is performed on GSEA 4.1.0 (http://www.broad.mit.edu/gsea/). Nominal *P*< 0.05 and FDR<0.25 were confirmed as statistical significance criteria. The infiltration scores of 16 immune cells and the activities of 13 immune-related functions were calculated by single-sample gene set enrichment analysis (ssGSEA) using the “GSVA” package. The expression of 47 immune checkpoint genes was calculated and demonstrated using the packages “limma”, “Reshape2” and “ggPUbr”.

### The value of the predictive signature in predicting UCEC chemotherapy response

To evaluate the predictive signature predicting treatment response to UCEC, we calculated the median maximal inhibitory concentration (IC50) for 138 common clinical chemotherapy drugs. Use “pRRophetic” package measuring IC50 value concentration between low risk and high risk group.

### Statistical analysis

R software (version 4.1.2) was used for all statistical analyses. The Wilcoxon test was used to analyze the expression levels of ER stress-related DEGs in cancer tissues and normal tissues. Univariate Cox regression analysis was used to analyze the correlation between ER stress-related lncrnas and overall survival (OS). Multivariate Cox analysis was used to screen ER stress-related lncrnas to establish predictive features. The Kaplan Meier method and sequential inspection analysis of low-risk and high-risk group of patients with OS. The receiver operating curve (ROC) was drawn by the “survivalROC” package and the area under the curve (AUC) was calculated.

## Results

### Enrichment analysis of ER stress-related genes

**Figure 1:**
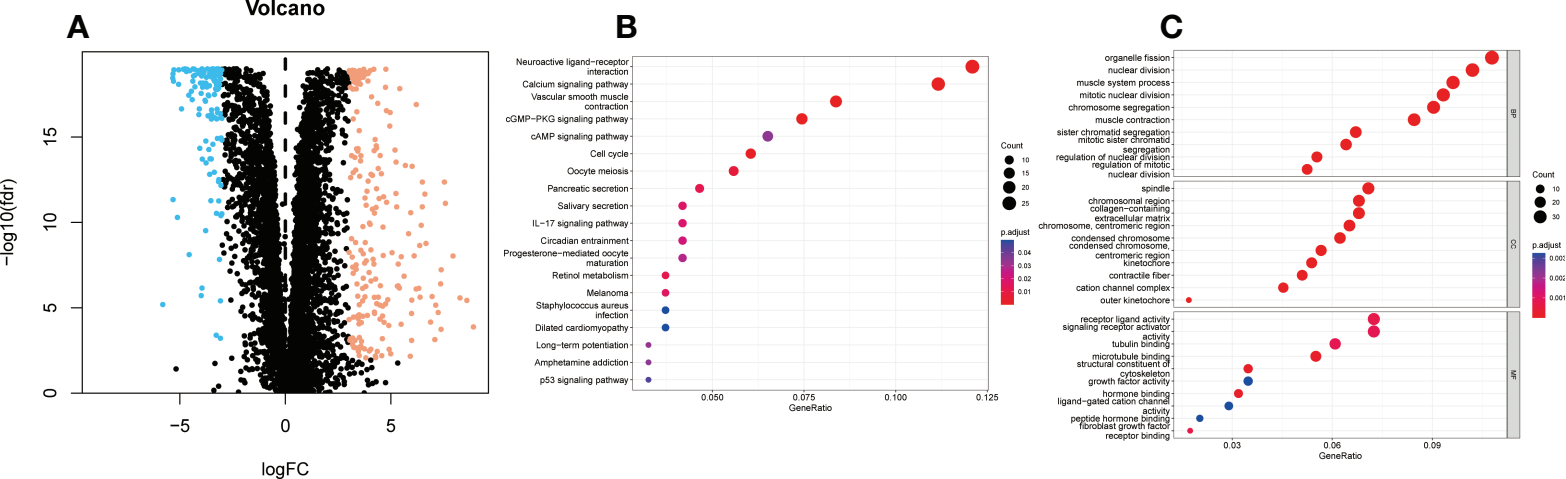
KEGG and GO analyses of endoplasmic reticulum stress related DEGs in UCEC and adjacent tissues. **(A)** Volcano plot of 8763 endoplasmic reticulum stress related genes in UCEC. Blue dots represent downregulated genes and yellow dots represent up-regulated genes. **(B)** KEGG analysis of endoplasmic reticulum stress -related DEGs. **(C)** GO analysis of endoplasmic reticulum stress related DEGs. KEGG, Kyoto Encyclopedia of Genes and Genomes; GO, Gene Ontology; DEGs, differentially expressed genes; fdr, false discovery rate; FC, fold change; BP, biological process; CC, cellular components; MF, molecular function.

We retrieved 359 ER stress-related DEGs, including 130 downregulated genes 229 upregulated genes and (**Figure 1A**). We performed KEGG and GO analyses of ER stress-related DEGs. KEGG pathway analyses represented that ER stress-related DEGs were predominantly enriched in Neuroactive ligand−receptor interaction and atherosclerosis, calcium signaling pathway, vascular smooth muscle contraction, cGMP−PKG signaling pathway, cAMP signaling pathway, cell cycle, the IL-17 signaling pathway, and p53 signaling pathway (**Figure 1B**). GO analysis indicated that DEGs were mainly enriched in organelle fission, nuclear division, muscle system process, spindle, chromosomal region, collagen−containing, receptor ligand activity, signaling receptor activator activity, and tubulin binding (**Figure 1C**).

### Establishment of the ER stress-related lncRNA predictive signature

**Figure 2:**
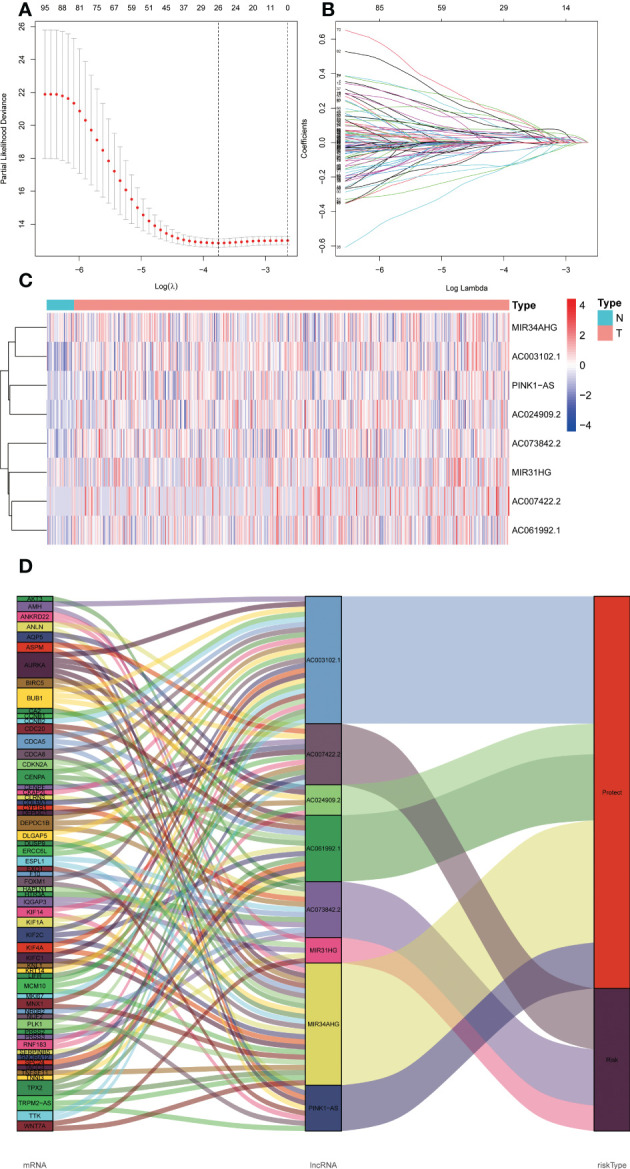
The expression levels and lncRNA-mRNA network of eight endoplasmic reticulum stress -related lncRNAs in the predictive signature. **(A, B)** Lasson regression establishes signature and divides TCGA dataset into training set and validation set. **(C)** The heatmap of expression levels with eight endoplasmic reticulum stress -related lncRNAs in UCEC and normal tissues. **(D)** Sankey diagram of prognostic endoplasmic reticulum stress -related lncRNAs. lncRNAs, long noncoding RNAs; UCEC, Uterine Corpus Endometrial Carcinoma; T, tumor; N, normal.

We acquired 1599 ER stress-related lncRNAs. The selection and construction of the Lasson regression are shown in **Figures 2A, B**. Univariate Cox regression analysis indicated that 103 lncRNAs were linked with the prognosis of UCEC patients. Multivariate Cox regression analysis showed that 8 ER stress-related lncRNAs (MIR34AHG, AC073842.2, PINK1AS, AC024909.2, MIR31HG, AC007422.2, AC061992.1, AC003102.1) were confirmed to establish a predictive signature. The expression levels of 8 ER stress-related lncRNAs in UCEC patients were shown in **Figure 2C**. Then, the “ggalluvial” R software package and Cytoscape were used to visualize the lncRNAs. The network included 198 pairs lncRNA-mRNA co-expression (**Figure 2D**, |R2 | > 0.3 and p<0.001). The risk score was estimated as follows: risk-score = (-0.833× MIR34AHG) + (0.760× AC073842.2) + (-1.005×PINK1AS) + (-0.885× AC024909.2) + (0.343×MIR31HG) + (0.396×AC007422.2) + (-0.399×AC061992.1) + (-0.727×AC003102.1).

### Correlation between the predictive signature and the prognosis of UCEC patients

**Figure 3:**
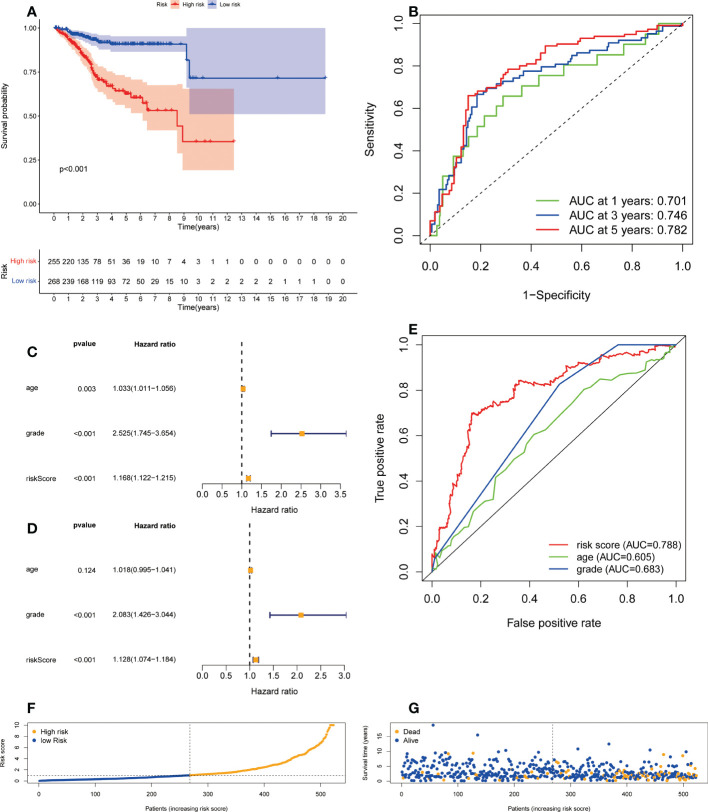
The correlation between the predictive signature and the prognosis of UCEC patients. **(A)** Kaplan-Meier analysis of the OS rate of UCEC patients between the high and low-risk groups. **(B)** The ROC curve and AUCs at one-year, three-years and five-years survival for the predictive signature. **(C)** Forest plot for univariate Cox regression analysis. **(D)** Forest plot for multivariate Cox regression analysis. **(E)** The ROC curve and AUCs of the risk score, age and grade. **(F)** The number of dead and alive patients with different risk scores. Blue represents the number of alive, and yellow represents the number of dead. **(G)** The distribution of the risk score in UCEC patients. UCEC, Uterine Corpus Endometrial Carcinoma; OS, overall survival; ROC, receiver operating curve; AUC, area under the curve.

**Figure 4:**
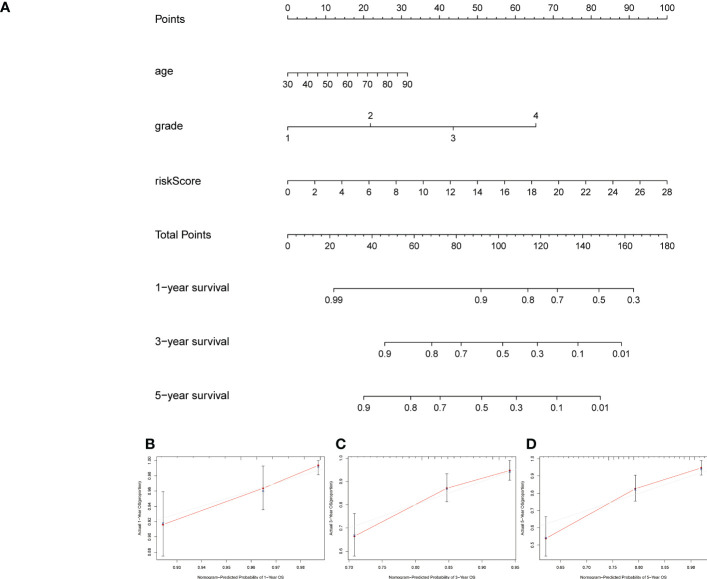
Creation and verification of the nomogram. **(A)** A nomogram integrating age, grade and risk score to predict 1-, 3-, and 5-year OS for UCEC patients. **(B–D)** Calibration curves tested the agreement between actual OS rates and predicted 1-, 3-, and 5-year survival rates. OS, overall survival.

A formula was used to calculate a risk score for each patient, and patients were divided into high-risk and low-risk groups according to the median risk score. Kaplan-meier analysis was used to determine the OS time in the high-risk and low-risk groups to assess the value of the risk score in predicting the prognosis of UCEC patients. The OS time of the high-risk group was significantly shorter than that of the low-risk group (**Figure 3A**, p<0.001). 1 year, 3 years and 5 years survival rate of AUC 0.701, 0.746 and 0.782, respectively, showed a favorable prediction effect (**Figure 3B**). Cox regression analysis was used to confirm whether the predictive characteristics were independent prognostic components in UCEC patients. Univariate Cox regression analysis showed that OS was significantly associated with age, grade, and risk score of UCEC patients (**Figure 3C**). Multivariate Cox regression analysis showed that OS was independently associated with grade and risk score of UCEC patients (**Figure 3D**). For prognosis prediction in UCEC patients, the AUC of the risk score was 0.788, which was superior to age and grade (**Figure 3E**). Low risk and high-risk groups of 5-year survival rates were 26.9% and 14.1% respectively. With the growth of the risk score, more and more UCEC patients died (**Figure 3F**). Low risk and high-risk group of risk score is shown in **Figure 3G**. We developed a nomogram including age, grade, and risk score to help predict the prognosis of UCEC patients. The 1 -, 3 -, and 5-year prognosis of UCEC patients can be predicted in this nomogram (**Figure 4A**). Calibration curve in the 1, 3, and 5-years survival rate and show the excellent performance between the actual rate of OS (**Figures 4B–D**).

### Relationship between the predictive signature and the prognosis of UCEC patients in age and grade

**Figure 5:**
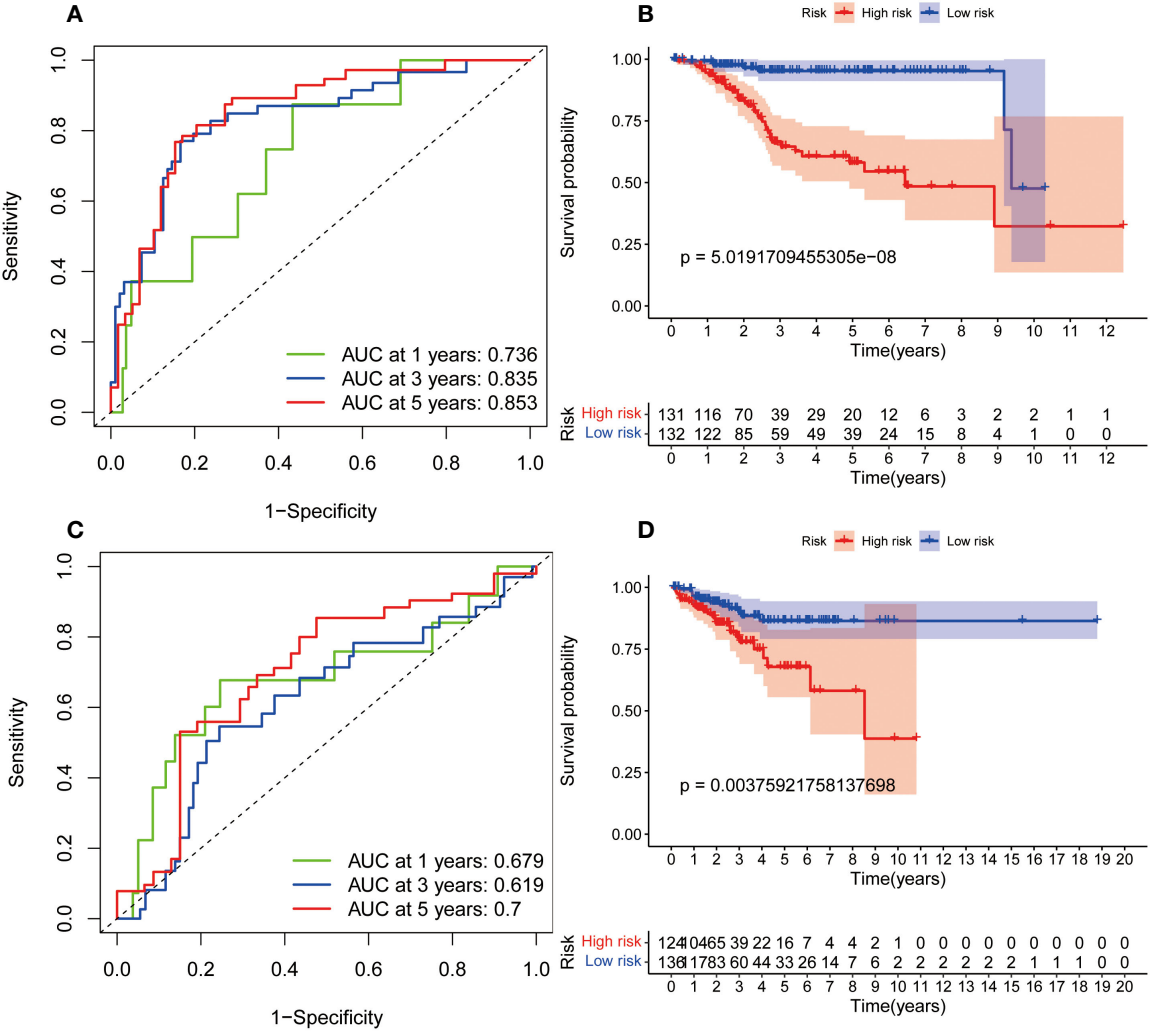
The internal verification of the predictive signature for OS relied on TCGA-UCEC data. **(A)** The ROC curve at 1-, 3-, and 5-year survival based on internal training cohort. **(B)** Kaplan-Meier survival curve in internal training cohort. **(C)** The ROC curve at 1-, 3-, and 5-year survival based on internal validation cohort. **(D)** Kaplan-Meier survival curve in internal validation cohort. ROC, receiver operating curve; AUC, area under the curve; OS, overall survival; TCGA, The Cancer Genome Atlas.

In order to investigate the relationship between the predictive signature and prognosis of patients in UCEC classified based on age and grade, UCEC patients were grouped into internal training cohort and validation cohort. The ROC curves of the two datasets demonstrated superior predictive value. In the internal training cohort, the AUCs of 1, 3, and 5-years survival were 0.736, 0.835, and 0.853, respectively (**Figure 5A**). The OS rate of UCEC patients in the high-risk group was worse than of the low-risk group in the internal training cohort (**Figure 5B**, *p=*5.02e-08). In the internal validation cohort, the AUCs of 1, 3, and 5-years survival were 0.679, 0.719, and 0.7 (**Figure 5C**). In the internal validation cohort, the prognosis of the high-risk group was lower than that of the low-risk group (**Figure 5D**, *p=*3.76e-03).

### Gene enrichment analysis

Because of the different outcomes of patients in the high-risk and low-risk groups, we performed GSEA to investigate possible differences between the high-risk and low-risk groups. We discovered that UCEC, cell cycle, fc gamma r mediated phagocytosis, Nod like receptor signaling pathway, pathways in cancer, oocyte meiosis, progesterone mediated oocyte maturation, ubiquitin mediated proteolysis, Notch signaling pathway, JAK-STAT signaling pathway, erbb signaling pathway. It suggests that high-risk patients are closely related to tumor and immune-related pathways (**Table 1**).

**Table 1:** The high-risk group enriched gene sets.

Gene set	ES	NES	NOM p-val	FDR q-val	FWER p-val
cell cycle	0.66	2.07	0.000	0.005	0.003
Fc gamma r mediated phagocytosis	0.65	2.07	0.000	0.003	0.004
Nod like receptor signaling pathway	0.61	1.96	0.000	0.012	0.026
pathways in cancer	0.50	1.88	0.000	0.022	0.083
oocyte meiosis	0.50	1.85	0.000	0.021	0.107
progesterone mediated oocyte maturation	0.52	1.83	0.006	0.026	0.139
ubiquitin mediated proteolysis	0.45	1.74	0.004	0.051	0.307
Notch signaling pathway	0.55	1.72	0.004	0.052	0.348
JAK-STAT signaling pathway	0.48	1.65	0.013	0.065	0.483
erbb signaling pathway	0.47	1.65	0.006	0.064	0.489

### Immune cell infiltration, immune-related pathways and immune checkpoint gene expression

**Figure 6:**
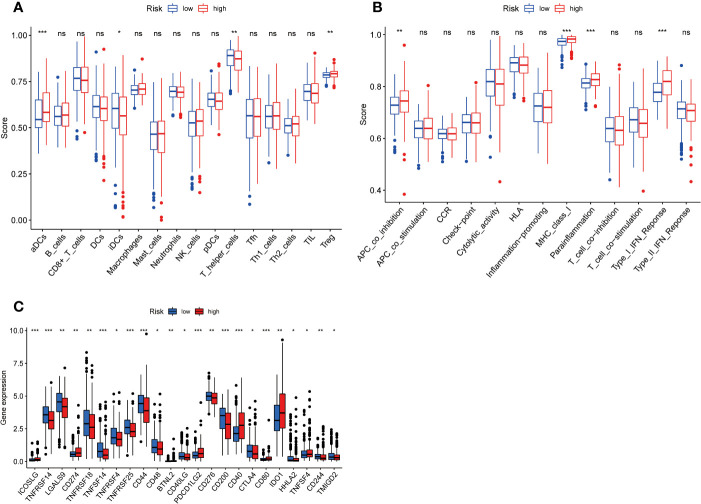
Figure 6 Immune infiltrating cell scores, immune-related functions, and immune checkpoint gene profiles in high-risk and low-risk populations. **(A)** Differences in infiltration of 16 immune cells between highrisk and low-risk groups were calculated using the ssGSEA algorithm. **(B)** Correlations of 13 immune-related functions with predictive signature in high- and low-risk populations. **(C)** Expression of immune checkpoint genes in high-risk and low-risk populations. ssGSEA, single sample gene set enrichment analysis; aDCs, activated dendritic cells; iDCs, immature dendritic cells; NK, natural killer; pDCs, plasmacytoid dendritic cells; Tfh, T follicularhelper; Th1, T helper type 1; Th2, T helper type 2; TIL, tumor-infiltrating lymphocyte; Treg, T regulatory cell; APC, antigen-presenting cell; CCR, chemokine receptor; HLA, human leukocyte antigen; MHC, major histocompatibility complex. *p < 0.05; **p < 0.01; ***p < 0.001; ns, non-significant.

To further clarify the connection between risk score and immune cells and immune function, we calculated ssGSEA enrichment scores for different immune cell subsets, related functions or pathways, and further investigated gene expression at immune checkpoints. The results demonstrated that activated dendritic cells (aDCs) and Treg were significantly increased in the high- groups, and immature dendritic cells (iDCs) and T helper cells were significantly decreased, compared with low- risk groups (**Figure 6A**). In the high-risk group, the immune function scores of APC co inhibition, MHC class I, para inflammation, type I IFN response were higher than the low-risk group (**Figure 6B**). These findings show that the immune function of high-risk groups is relatively more active. In addition, we also found significant differences in immune checkpoint genes CD274 (PD-L1), BTNL2, CD40, CD80, IDO1 and TNFSF4 among high-risk populations (**Figure 6C**). The expression of PD-L1 in the high-risk group was significantly higher than that in the low-risk group, indicating that high-risk patients may be effective in anti-PD-1/L1 immunotherapy.

### Relationship between the predictive signature and UCEC therapy

**Figure 7:**
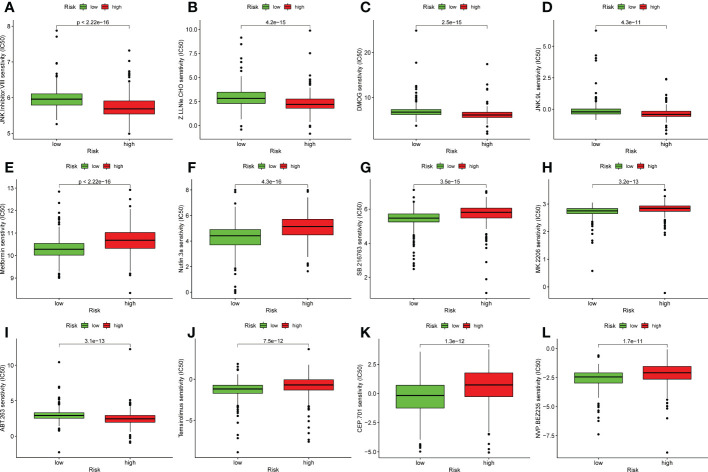
Comparison of susceptibility to twelve therapeutic agents between high-risk and low-risk groups. **(A)** IC50 of JNK inhibitor VIII high and low risk groups. **(B)** IC50 of Z.LLNle.CHO high and low risk groups. **(C)** IC50 of DMOG in high and low risk groups. **(D)** IC50 of JNK.9L in high and low risk groups. **(E)** IC50 of Metformin in high and low risk groups. **(F)** IC50 of Nutlin.3a in high and low risk groups. **(G)** IC50 of SB.216763 in high and low risk groups. **(H)** IC50 of MK.2206 in high and low risk groups. **(I)** IC50 of ABT.263 in high and low risk groups. **(J)** IC50 of Temsirolimus in high and low risk groups. **(K)** IC50 of CEP.701 in high and low risk groups. **(L)** IC50 of NVP.BEZ235 in high and low risk groups.

In addition to immunotherapy, we also analyzed the association between the predictive signature and the efficacy of general chemotherapy for UCEC. In addition, we analyzed the association between predicted signature and the efficacy of conventional UCEC chemotherapy agents. The results showed that, compared with the low-risk group, the IC50 of JNK inhibitor VIII, Z.LLNle.CHO, DMOG and JNK.9L were lower (**Figures 7A–D**) and, the IC50 of Metformin, Nutlin.3a, SB.216763, MK.2206, ABT.263, Temsirolimus, CEP.701, and NVP.BEZ235 in the high-risk group was higher (**Figures 7E–L**), which is conducive to the formulation of precise treatment plan suitable for the high-and low-risk population.

### Establishment of the ER stress-related lncRNA predictive signature for DFS

**Figure 8:**
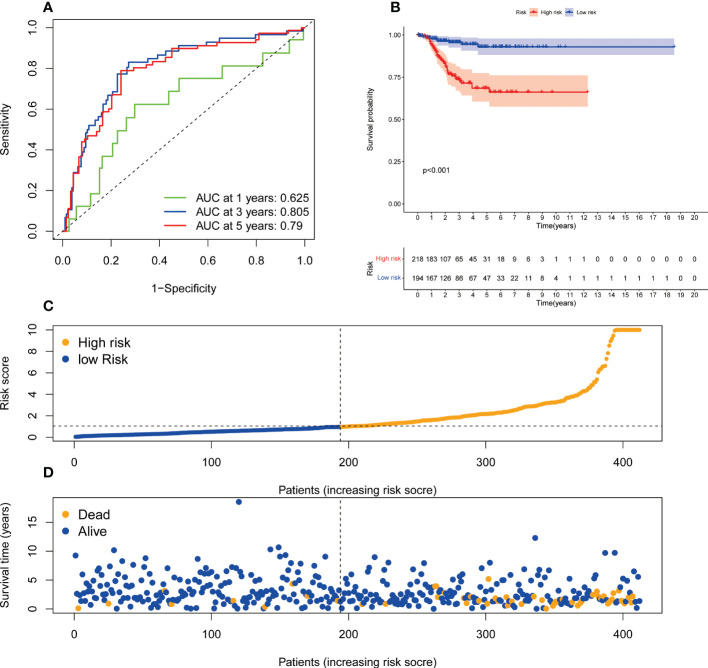
The predictive value of endoplasmic reticulum stress -related lncRNA signature in DFS. **(A)** ROC curve at 1-, 3-, and 5-year survival based on TCGA-UCEC data. **(B)** Kaplan-Meier survival curve based on TCGA-UCEC data. **(C)** The number of dead and alive patients with different risk scores. Blue represents the number of alive, and yellow represents the number of dead. **(D)** The distribution of the risk score in UCEC patients. DFS, disease-free survival; ROC, receiver operating characteristic; AUC, area under the curve.

We also established the predictive signature of ER stress-related lncRNA in DFS to further clarify the role of DFS in the prognosis of patients in UCEC. We collected the DFS data of UCEC patients from the cBioPortal database, including 529 patients. Univariate Cox regression analysis showed that 27 ER stress-related lncRNAs were significantly associated with DFS in UCEC. Multivariate Cox regression analysis demonstrated that 7 ER stress-related lncRNAs were confirmed to establish the predictive signature. Risk score= (-1.659× AC004943.2) + (-0.675×ZBED5-AS1) + (0.465×AC011477.3) + (1.287× HMGN3-AS1) + (0.707×AC132872.3) + (-0.302×LBX2-AS1) + (0.729×WASIR2). The patients in the whole dataset were calculated based on the formula for each patient’s risk score, and the patients were divided into high-risk group and low-risk group by referring to the median risk score. The AUCs of 1, 3, and 5-years survival were 0.625, 0.805, and 0.79, respectively (**Figure 8A**). Kaplan-Meier survival curve analysis indicated that DFS was significantly shorter in the high-risk group than in the low-risk group (**Figure 8B**, *p*<0.001). With the growth of risk score, more and more UCEC patients died (**Figure 8C**). The risk scores of the low-risk and high-risk groups are demonstrated in **Figure 8D**.

## Discussion

The most common gynecological malignancy is uterine corpus endometrial carcinoma (UCEC), which accounts for about 90% of cases (14). It is difficult to determine how ER stress manifests in gynecological tumors. It has been shown that cancer is closely related to genes associated with ER stress (15). Therefore, targeting genes that are associated with ER stress is essential for treating cancer (16). The prognostic value of its use in gynecological tumors has been predominantly unstudied. Several studies have developed lncRNAs associated with ER stress that predict prognosis in cancer patients (11,17,18). In breast cancer patients with high risk of poor outcomes, CAI et al. identified nine ERS-associated lncRNAs (11). According to a previous study, eight ER stress-related lncrnas can be used as prognostic indicators for colorectal adenocarcinoma (17). A Cox regression analysis was performed to determine risk signature in glioma based on six ER stress-related lncRNAs, and LINC00519 silencing inhibited glioma cell migration and invasion (18). Therefore, ER stress-related lncRNAs play an essential role in the diagnosis, treatment and prognosis of tumors. Despite this, ER stress-related lncRNAs have not yet been established as prognostic factors for UCEC patients.

A total of 359 DEGs related to ER stress were obtained in this study. According to KEGG analysis, Neuroactive ligand−receptor interaction and atherosclerosis, calcium signaling pathway, vascular smooth muscle contraction, cGMP−PKG signaling pathway, cAMP signaling pathway, cell cycle, the IL-17 signaling pathway, and p53 signaling pathway are involved in UCEC. An analysis of GO symbols indicated that DEGs were mainly enriched in organelle fission, nuclear division, muscle system process, spindle, chromosomal region, collagen−containing, receptor ligand activity, signaling receptor activator activity, and tubulin binding. Immunotherapy, chemotherapy, targeted therapy, and tumor growth are all affected by abnormal activation of ER stress sensors and their downstream signaling pathways (19). The ER separates, stores and releases calcium in cells, and is involved in regulating gene transcription in the nucleus, energy metabolism in mitochondria and cytoplasmic signaling pathways (20). As a result of preventing phosphodiesterase-2, dipyridamole enhances ER stress-induced NoXA-guided apoptosis through an increase in intracellular cAMP levels (21). Blocking the interaction between IL-17A and ER stress attenuates LPS-induced lung injury (22). When combined with mutant p53 targets, ATF6 inhibitors can increase the sensitivity of cancer cells to endogenous or chemotherapy-induced ER stress (23). However, additional research is required to confirm the function of ER stress-related genes in UCEC. Many studies have shown that lncRNAs in UCEC play an important role. Clinical Outcome prediction of Endometrial Carcinoma based on a 3-lncRNA Signature (24).Potential biomarkers for predicting prognoses and immunological responses in UCEC patients include pyroptosis-related lncRNAs (14). Therefore, it is necessary to develop and verify the prognostic signature of ER stress-related IncRNA in UCEC patients.

In this study, univariate Cox regression analysis was used to analyze the relationship between ER stress-related lncRNAs and the prognosis of UCEC patients, and 103 lncRNAs were found to be associated with the prognosis of UCEC patients. After multivariate Cox regression analysis, eight lncRNAs (MIR34AHG, AC073842.2, PINK1AS, AC024909.2, MIR31HG, AC007422.2, AC061992.1, AC003102.1) associated with ER stress were identified to be included in the prediction signature. Survival rates of AUC 0.701, 0.746, and 0.782 for 1 year, 3 years, and 5 years, respectively, showed superior prediction ability. Researchers have identified seven lncRNAs as potential prognostic factors for UCEC, and the area under the curve (3-year survival rate) is 0.797 (25). As far as diagnostic efficacy is concerned, their results are comparable to ours.

According to the formula to calculate the risk score for each patient. Patients were divided into high-risk and low-risk groups based on median values. The high-risk group had a shorter OS time than the low-risk group. The ROC curve indicates that the predictive signature has good predictive value. The predictive signature was more reliable than clinic pathological variables in predicting the prognosis of UCEC patients. Signature of prognosis in patients with UCEC forecast more reliable than the clinical pathological indicators. Moreover, we also found that predictive features could predict the prognosis of UCEC patients regardless of clinicopathological factors. The internal verification proves that the predictive signature has excellent predictive performance value. Different types of tumors are associated with genes involved in endoplasmic reticulum stress. In the case of hepatocellular carcinoma, the prediction model based on endoplasmic reticulum-related genes showed superior performance (26, 27). A surprising association was also found between endoplasmic reticulum stress-related genes and drug therapy in Clear cell renal cell carcinoma (28). Therefore, UCEC patients’ prognosis was affected by the endoplasmic reticulum stress-related gene IncRNA signature as well.

GSEA showed that cell cycle, fc gamma r mediated phagocytosis, Nod like receptor signaling pathway, pathways in cancer, oocyte meiosis, progesterone mediated oocyte maturation, ubiquitin mediated proteolysis, Notch signaling pathway, JAK-STAT signaling pathway, erbb signaling pathway were mainly enriched in the high-risk group. PHE exposure induced mitochondrial dysfunction and ER stress, which results in the cytoplasm reactive oxygen species and abnormal calcium levels, final oocyte induced oxidative stress and DNA damage (29). Genes related to ER stress are associated with multiple immune pathways. The association of NOD1 and NOD2 with proinflammatory responses induced by IRE1α/TRAF2 signaling provides a novel link between innate immunity and ER stress-induced inflammation (30). The activation of UPR caused by ER stress may help to correct the increased level of GLP-1/Notch signaling and the associated overproliferation in the germ line of C. elegans (31). The ss-GSEA results demonstrate a significant relationship between the prediction signatures and dendritic cell activation, immature dendritic cells, immune status of the Treg, and dendritic cell activation. A high-risk group may be more sensitive to PD – 1/L1 immunotherapy and JNK inhibitors VIII, Z.LLNle.CHO, DMOG. The four immune-related lncRNAs were extremely effective in the overall OS prognosis of UCEC, which was similar to our study design (32). It was nevertheless possible for them to distinguish patients from different UCECs despite the NMF algorithm they employed to classify the population. There were, however, individualized medication choices available to people with different risk scores.

It is significant to note, however, that our study has several limitations. As a first step, we only verified the data from the TCGA database, but we also needed data from other databases to verify the predictive signature’s applicability. In addition, further experiments need to be conducted to confirm how ER stress-related lncRNAs function in UCEC.

A lncRNA signature related to ER stress can independently predict the prognosis of patients with UCEC. UCEC tumor immune microenvironment was characterized by the activation of aDCs, Tregs, and iDCs. There may be an association between poor prognoses and immune function conditions such as co-inhibition of APC, MHC class I, para-inflammatory conditions, and type I IFN. There is, however, something intriguing about the fact that high-risk patients with UCEC can select chemotherapy and immunotherapy separately based on their risk level. In a word, UCEC and their response to clinical therapeutic drugs can be explained in part by ER stress-related lncRNAs, although additional experimental verification is required.

## Data availability statement

The datasets presented in this study can be found in online repositories. The names of the repository/repositories and accession number(s) can be found in the article/supplementary material.

## Author contributions

YY and JC contributed conception and design of the study; LS and YY collected the data; JC and LS performed the statistical analysis; JC wrote the first draft of the manuscript; LS and YY revised the manuscript; YY gave the final approval of the version to be submitted. All authors contributed to manuscript and approved the submitted version.
